# Hypoxic adipose‐derived stem cell exosomes as carriers of miR‐100‐5p to enhance angiogenesis and suppress inflammation in diabetic foot ulcers

**DOI:** 10.1002/ccs3.70018

**Published:** 2025-06-27

**Authors:** Hong Liu, Fei Hao, Bangtao Chen

**Affiliations:** ^1^ Dermatology and Plastic Surgery Center The Third Affiliated Hospital of Chongqing Medical University Chongqing China; ^2^ Department of Orthopedics Chongqing University Three Gorges Hospital Chongqing China; ^3^ Chongqing Municipality Clinical Research Center for Geriatric Diseases Chongqing China; ^4^ Department of Dermatology Chongqing University Three Gorges Hospital School of Medicine Chongqing University Chongqing China

**Keywords:** adipose‐derived stem cells, angiogenesis, cell signaling, diabetic foot ulcer, hypoxic exosomes, inflammation, miR‐100‐5p

## Abstract

Diabetic foot ulcer (DFU) is a severe diabetes complication characterized by impaired angiogenesis and chronic inflammation, leading to delayed wound healing. Exosomes (Exo) derived from hypoxic adipose‐derived stem cells (H‐ADSCs‐Exo) show potential as therapeutic carriers. This study investigates the role of H‐ADSCs‐Exo carrying miR‐100‐5p in DFU healing. ADSCs were isolated, characterized, and their Exo analyzed via transmission electron microscopy, nanoparticle tracking analysis, and Western blot. Transcriptome sequencing identified miR‐100‐5p as a key modulator of angiogenesis and inflammation. In vitro, H‐ADSCs‐Exo enhanced human umbilical vein endothelial cell and fibroblast proliferation, migration, and tube formation. In a rat DFU model, H‐ADSCs‐Exo administration reduced ulcer size, increased angiogenesis (VEGF/CD31 expression), and decreased inflammatory markers (TNF‐α, IL‐6). miR‐100‐5p overexpression further amplified these effects, demonstrating its critical role in Exo‐mediated healing. These findings highlight the therapeutic potential of H‐ADSCs‐Exo in DFU treatment, offering insights into cell signaling mechanisms and paving the way for miRNA‐based regenerative therapies.

## Graphical abstract

This study investigates the therapeutic potential of hypoxic adipose‐derived stem cell exosomes (H‐ADSCs‐Exo) carrying miR‐100‐5p in diabetic foot ulcer (DFU) healing. Findings show that H‐ADSCs‐Exo enhance angiogenesis, suppress inflammation, and accelerate wound healing in vitro and in vivo. miR‐100‐5p plays a pivotal role in these effects, offering a novel strategy for DFU treatment.

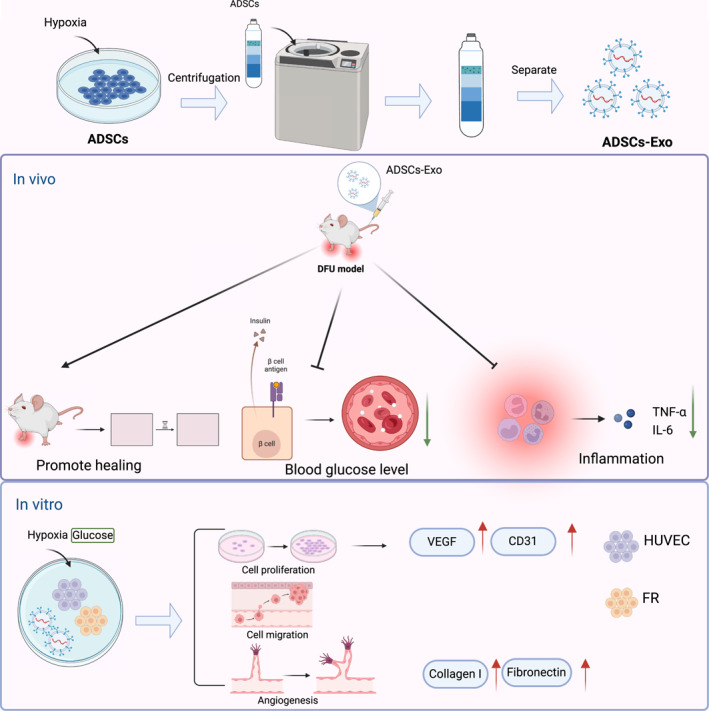

## 1 INTRODUCTION

Diabetic foot ulcer (DFU) is one of the most common and severe complications of diabetes, often leading to serious outcomes such as infection, hospitalization, lower‐limb amputation, and even death.^1^ The development of DFU is closely linked to poor long‐term glycemic control, which results in impaired microcirculation, neuropathy, and reduced angiogenesis.^2^ These pathological changes delay wound healing, increasing the risk of infection and recurrence.^3^ Clinical data indicate that the 5‐year survival rate of patients with DFU is lower than that of some patients with cancer, highlighting its severe impact on health and life expectancy.^4^ Although current treatments, including medications, wound care, surgical interventions, and biological therapies, are available, their effectiveness remains suboptimal, significantly diminishing patients' quality of life.^5^ Therefore, there is an urgent need to develop new therapeutic strategies, particularly those that can effectively promote angiogenesis and reduce inflammatory response (IR).

Although existing DFU treatments provide some benefits, their overall efficacy is limited. Pharmacological therapies primarily focus on glycemic control and infection management,^6^ yet they fail to effectively improve local microcirculation and angiogenesis.^7^, ^8^, ^9^ Although surgical interventions and wound‐healing materials can accelerate ulcer recovery, their success largely depends on the patient's overall health and diabetes management.^10^ Research has identified chronic IRs in DFUs as one of the key factors hindering wound healing, and current anti‐inflammatory therapies do not fully address this issue. Regenerative medicine has started to reveal new pathways for tissue repair, such as stem cell therapy, gene therapy, and bioactive materials, but the clinical application of these approaches is still in its early stages^11^ and requires further research and optimization.

In recent years, exosomes (Exo) have gained increasing attention in the scientific community as key mediators of intercellular communication. Exo are small vesicles secreted by cells, rich in proteins, lipids, and miRNAs, which can regulate the function of recipient cells. Studies have shown that Exo play a critical role in tissue repair and regeneration, particularly in promoting angiogenesis, modulating immune responses, and reducing inflammation.^12^, ^13^, ^14^ Adipose‐derived stem cell exosomes (ADSCs‐Exo) have emerged as potential tools for treating DFU due to their accessibility, low immunogenicity, and strong tissue repair capabilities.^15^ Previous research has demonstrated that ADSCs‐Exo can accelerate wound healing and regulate local tissue regeneration through the bioactive molecules they carry, such as miRNAs.^16^ Therefore, using Exo for tissue repair and the treatment of DFU is a promising research direction.

Hypoxia significantly influences cellular metabolism and alters Exo composition and function. Studies suggest that hypoxic adipose‐derived stem cell exosomes (H‐ADSCs‐Exo) exhibit enhanced capabilities in regulating angiogenesis and IRs.^17^, ^18^ Specifically, H‐ADSCs‐Exo have been shown to promote angiogenesis by stimulating endothelial cell proliferation and migration while simultaneously suppressing IRs, thereby improving tissue healing capacity.^19^, ^20^ Among the bioactive components of these Exo, miR‐100‐5p has been identified as a key regulator of both angiogenesis and IRs. By targeting multiple signaling pathways, miR‐100‐5p plays a crucial role in maintaining tissue microenvironment homeostasis. Consequently, H‐ADSCs‐Exo carrying miR‐100‐5p hold significant potential as a therapeutic intervention for DFU.

This study aims to investigate the mechanisms by which miR‐100‐5p, delivered by H‐ADSCs‐Exo, promotes the healing of DFUs. Through both in vitro and in vivo experiments, we analyzed the regulatory effects of H‐ADSCs‐Exo on angiogenesis, IRs, and various biological processes such as cell proliferation and migration. The results demonstrated that H‐ADSCs‐Exo not only accelerated the healing of foot ulcers but also significantly enhanced the angiogenic capacity of endothelial cells and reduced local inflammation. This study offers new insights and potential therapeutic targets for the treatment of DFUs, particularly highlighting the promise of Exo‐ and miRNA‐based personalized treatment strategies, which hold the potential for breakthrough clinical applications in the future. Moreover, this research contributes a valuable scientific understanding of the pathological mechanisms underlying DFUs and provides a solid foundation for the development of novel biotherapeutic approaches.

## 2 MATERIALS AND METHODS

### 2.1 Isolation and purification of ADSCs

Inguinal white adipose tissue (iWAT) was harvested from the subcutaneous abdominal region of adult SD rats (purchased from Beijing Vital River Laboratory Animal Technology Co. Ltd., strain code: 101). The tissue was minced under sterile conditions and digested with 0.1% collagenase I (C0130, Sigma‐Aldrich, USA) at 37°C for 45 min. The resulting cell suspension was filtered through a 200‐mesh sieve (pluriSelect, Germany) to remove undigested tissue fragments. After filtration, the suspension was centrifuged at 1200 rpm for 5 min, and the supernatant was discarded. The cell pellet was resuspended in a culture medium containing 10% fetal bovine serum (FBS, 26140079, Gibco, USA), 1% penicillin‐streptomycin (15140122, Gibco, USA), and high‐glucose DMEM (11965092, Gibco, USA). The cells were seeded into culture flasks and incubated at 37°C in a 5% CO_2_ atmosphere. After cell adhesion, the medium was replaced to remove nonadherent cells. When the cells reached 80%–90% confluence, they were passaged using 0.25% trypsin (25200056, Gibco, USA).

### 2.2 Identification of ADSCs

Flow cytometry (BD FACSCanto II, BD Biosciences, USA) was performed to evaluate the purity and characteristics of ADSCs based on surface marker expression. Adherent ADSCs were harvested, washed twice with PBS, and resuspended in PBS containing 0.1% BSA (A3294, Sigma‐Aldrich, USA). Cells were incubated at 4°C for 30 min with specific antibodies targeting CD29 (555005, BD Biosciences, USA), CD44 (550974, BD Biosciences, USA), CD90 (554895, BD Biosciences, USA), CD73 (ANT‐066‐200UL, Alomone Labs, USA), CD34 (PA5‐85917, Invitrogen, USA), CD19 (CD19‐FITC, FabGennix, USA), and CD45 (11‐0461‐82, eBioscience, USA), followed by three washes with PBS. Flow cytometry analysis was performed in triplicate. To further verify the multipotency of ADSCs, cells were cultured in adipogenic differentiation medium (PT‐8002, Lonza, USA) for 14 days, followed by Oil Red O staining (O0625, Sigma‐Aldrich, USA), or in osteogenic differentiation medium (PT‐3002, Lonza, USA) for 9 days, followed by alkaline phosphatase staining (SCR004, Sigma‐Aldrich, USA).

### 2.3 Isolation of ADSCs‐Exo

ADSCs‐Exo were isolated using a combination of differential centrifugation and ultracentrifugation (UC). The ADSC culture supernatant was first centrifuged at 300 g for 10 min to remove cells and debris. The resulting supernatant was then centrifuged at 2000 g for 20 min to eliminate larger debris and microvesicles. Next, the supernatant was centrifuged at 10,000 g for 30 min to obtain a partially purified Exo suspension. Using an ultracentrifuge (Optima XPN‐100, Beckman Coulter, USA), the suspension was further centrifuged at 100,000 g for 70 min. The supernatant was discarded, and the Exo was resuspended in PBS, followed by another round of centrifugation under the same conditions. The final Exo pellet was resuspended in 200 μL of PBS. All experiments were performed in triplicate.

### 2.4 Characterization of ADSCs‐Exo

The morphology and size distribution of ADSCs‐Exo were characterized using transmission electron microscopy (TEM) (Tecnai G2 Spirit BioTWIN, FEI, USA) and nanoparticle tracking analysis (NTA) (NanoSight NS300, Malvern Panalytical, UK). Purified ADSCs‐Exo suspensions were placed onto copper grid slides and dried at 4°C for 30 min. The samples were then negatively stained with 2% phosphotungstic acid (195651, Sigma‐Aldrich, USA) and air‐dried at room temperature before TEM imaging. To determine particle size and concentration, diluted Exo suspensions were analyzed using NTA. All characterization experiments were performed in triplicate.

### 2.5 Western blot (WB)

Cells or Exo were lysed using RIPA lysis buffer (P0013B, Beyotime, China), and protein concentrations were quantified using the BCA assay (23225, Thermo Fisher Scientific, USA). Protein samples were separated by SDS‐PAGE and transferred onto PVDF membranes (Millipore, USA), followed by blocking with 5% skim milk for 1 h. The membranes were incubated overnight at 4°C with the following primary antibodies: CD63 (PA5‐100713, 1:1000, Invitrogen, USA), CD81 (ab109201, 1:1000, Abcam, UK), TSG101 (ab125011, 1:1000, Abcam, UK), vascular endothelial growth factor (VEGF) (ab214424, 1:1000, Abcam, UK), angiopoietin 1 (ab183701, 1:1000, Abcam, UK), collagen I (ab260043, 1:1000, Abcam, UK), and fibronectin (ab45688, 1:1000, Abcam, UK). The next day, the membranes were washed three times with TBST, each for 10 min, and incubated with HRP‐conjugated secondary antibody (ab6721, 1:5000, Cell Signaling Technology, USA) for 1 h at room temperature. Protein bands were visualized using an enhanced chemiluminescence (ECL) reagent (32106, Thermo Fisher Scientific, USA) and detected with a gel imaging system (Bio‐Rad, USA). All experiments were performed in triplicate.

### 2.6 Download of DFU‐Related data

DFU‐related transcriptome data were retrieved from the Gene Expression Omnibus (GEO) database (https://www.ncbi.nlm.nih.gov/geo/), specifically dataset GSE68184. For bioinformatics analysis, three DFU skin samples were selected as the experimental group, whereas three nondiabetic foot ulcer (NFU) (healthy) skin samples were used as controls.

### 2.7 miRNA extraction and quality assessment

miRNA was extracted from purified ADSCs‐Exo using the mirVana miRNA Isolation Kit (Ambion, USA) following the manufacturer's instructions. The concentration of the extracted miRNA was measured using a NanoDrop 2000 Spectrophotometer (Thermo Fisher Scientific, USA), whereas RNA integrity and quality were assessed with an Agilent 2100 Bioanalyzer (Agilent Technologies, USA), ensuring an RNA integrity number (RIN) greater than 7.0.

### 2.8 miRNA library construction and sequencing

The miRNA library was prepared using the TruSeq Small RNA Sample Prep Kit (Illumina, USA). Sequencing was performed on the Illumina HiSeq 2500 platform (Illumina, USA) with a single‐end 50 bp read length, generating over 20 million high‐quality reads per sample.

### 2.9 Data preprocessing

Raw sequencing reads were processed using Cutadapt v2.10 to remove adapter sequences and low‐quality bases. The cleaned reads underwent quality control assessment using FastQC v0.11.9 to ensure data integrity.

### 2.10 Differential expression analysis

Differential expression analysis was conducted using DESeq2 v1.30.1, applying a significance threshold of *p* < 0.05 and |log_2_ fold change| >1. The identified differentially expressed miRNAs were further analyzed. The VennDiagram package in R software was used to determine the intersection of relevant genes, and a Venn diagram was generated to visualize overlapping genes.

### 2.11 GO/KEGG functional enrichment analysis

The predicted target genes were subjected to Gene Ontology (GO) and Kyoto Encyclopedia of Genes and Genomes (KEGG) pathway enrichment analysis using clusterProfiler v3.18.1, with the significance threshold set at *p* < 0.05. The results were visualized for further interpretation.

### 2.12 LASSO regression algorithm

The least absolute shrinkage and selection operator (LASSO) regression algorithm was applied to identify key disease‐related genes. To ensure reproducibility, a random seed was set, and the glmnet package was used to analyze the dataset containing multiple variables. The glmnet function was employed to perform LASSO regression on selected candidate genes, modeling the data as a binary classification problem. A regular expression was used to extract sample class labels as response variables. Model evaluation was conducted by plotting the model object and using cross‐validation with the cv.glmnet function to determine the optimal lambda value. Genes corresponding to nonzero coefficients at the optimal lambda were identified as key genes associated with disease status. This approach not only enabled precise gene selection but also enhanced predictive accuracy by reducing model overfitting, providing strong support for biomarker discovery and disease mechanism research.

### 2.13 Isolation of H‐ADSCs‐Exo

ADSCs were cultured under 1% O_2_ conditions to induce hypoxia. Cells were incubated for 48 h in a Hypoxia Incubator Chamber (STEMCELL Technologies, Canada) at 37°C, with 5% CO_2_ and 1% O_2_. After hypoxic treatment, H‐ADSCs‐Exo were isolated and purified using differential centrifugation and UC. The purified Exo were characterized using TEM, NTA, and WB. All experiments were performed in triplicate.

### 2.14 Establishment of the DFU rat model

Eight‐week‐old male SD rats (200–220 g, purchased from Beijing Vital River Laboratory Animal Technology Co. Ltd., strain code: 101) were housed under standard conditions (temperature 22 ± 2°C, 12‐h light/dark cycle, with free access to food and water) for 1 week to acclimate. Diabetes was induced by intraperitoneal injection of streptozotocin (STZ, S0130, Sigma‐Aldrich, USA) at a dose of 60 mg/kg body weight. Blood glucose levels were monitored on the 3rd and 7th days postinjection, and rats with blood glucose levels >16.7 mmol/L were considered successfully diabetic. To establish the DFU model, rats were anesthetized with isoflurane, and an 8 mm diameter circular ulcer was created on the hind foot. Postoperatively, dressings were changed daily, and ulcer size was recorded using digital photography. The success of the model was assessed through visual inspection.^21^, ^22^

### 2.15 Cell culture

In vitro experiments were conducted using human umbilical vein endothelial cells (HUVEC, CRL‐1730, ATCC, USA) and rat skin fibroblasts (FR, CRL‐1213, ATCC, USA). The cells were cultured in high‐glucose DMEM (11965092, Gibco, USA) supplemented with 10% FBS (26140079, Gibco, USA) and 1% penicillin‐streptomycin (15140122, Gibco, USA). Cells were maintained in an incubator at 37°C with 5% CO_2_. To simulate a diabetic environment, D‐glucose (G7021, Sigma‐Aldrich, USA) was added to the medium to achieve a final concentration of 25 mM.

### 2.16 CCK‐8 cell proliferation assay

Cell proliferation was assessed using the cell counting kit‐8 (CCK‐8) (CCK‐8, CK04, Dojindo, Japan). HUVEC and FR cells were seeded into 96‐well plates at a density of 5000 cells per well. After adding Exo from different treatment groups, 10 μL of CCK‐8 solution was added to each well at 0, 24, 48, and 72 h. After incubation at 37°C for 2 h, absorbance was measured at 450 nm using a microplate reader. All experiments were performed in triplicate.

### 2.17 Transwell migration assay

Cell migration was assessed using Transwell chambers (8 μm pore size, 352180, Corning, USA). HUVEC and FR cells were seeded in the upper chamber at a density of 1 × 10^5^ cells per well, whereas the lower chamber was filled with culture medium containing 10% FBS. After 24 h of incubation, nonmigrated cells in the upper chamber were removed, whereas the migrated cells in the lower chamber were fixed and stained. The migrated cells were observed and counted under a microscope (Olympus IX71, Japan). All experiments were performed in triplicate.

### 2.18 Tube formation assay

To assess angiogenesis, 50 μL of Matrigel (354230, BD Biosciences, USA) was added to a 96‐well plate and solidified at 37°C. HUVEC were seeded on the Matrigel at a density of 1 × 10^4^ cells per well, followed by the addition of Exo from different treatment groups. After 6 h of incubation, tube‐like structures were observed and photographed under a microscope (Olympus IX71, Japan). The number, length, and junctions of tube formation were measured using ImageJ software. All experiments were repeated three times.

### 2.19 Uptake of ADSCs‐Exo

To analyze Exo uptake, normoxic ADSCs‐Exo (N‐ADSCs‐Exo) and H‐ADSCs‐Exo were fluorescently labeled using PKH26 dye (PKH26GL, Sigma‐Aldrich, USA). Labeled ADSCs‐Exo were then coincubated with HUVEC and FR cells for 6 h, and their uptake was observed using confocal microscopy (Leica TCS SP8, Germany), with the experiment repeated three times.

### 2.20 Cell transfection and grouping

ADSCs were transfected with lentiviral vectors (GeneChem, China) to overexpress miR‐100‐5p mimic and NC mimic (control). The transfection was performed at an MOI of 10, following the manufacturer's instructions. After transfection, the cells were cultured for 72 h in high‐glucose DMEM containing 10% FBS (11965092, Gibco, USA), and positive cells were selected using puromycin (2 μg/mL, P8833, Sigma‐Aldrich, USA). Additionally, miR‐100‐5p inhibitor (miR20000098‐1‐5, RIBOBIO) and NC inhibitor (control) (miR2N0000001‐1‐5, RIBOBIO) were transfected into ADSCs, and transfection efficiency was verified by quantitative real‐time polymerase chain reaction (RT‐qPCR).

### 2.21 Validation of transfection efficiency by RT‐qPCR

Total RNA was extracted from transfected ADSCs using TRIzol reagent (15596018, Invitrogen, USA). RT‐qPCR was performed using the TaqMan MicroRNA Assay (4427975, Applied Biosystems, USA), with U6 serving as the internal control. The reaction setup and protocol followed the manufacturer's instructions, and amplification was carried out using the ABI 7500 Real‐Time PCR System (Applied Biosystems, USA). Relative expression levels were calculated using the 2^−ΔΔCt^ method, with primer sequences provided in Table S1.

### 2.22 Immunofluorescence staining

To observe cytoskeletal reorganization and angiogenesis, immunofluorescence staining was performed. Treated HUVEC were fixed on glass slides using 4% formaldehyde (F8775, Sigma‐Aldrich, USA) for 15 min. The cells were then permeabilized with 0.1% Triton X‐100 (T8787, Sigma‐Aldrich, USA) for 10 min. After blocking with 5% BSA (A3294, Sigma‐Aldrich, USA) for 30 min, the slides were incubated overnight at 4°C with primary antibodies against F‐actin (ab205, 1:200, Abcam, UK) and VE‐cadherin (ab33168, 1:200, Abcam, UK). The next day, the slides were washed with PBS and incubated for 1 h at room temperature with secondary antibodies: goat anti‐mouse IgG H&L (Alexa Fluor® 488) (ab150113, 1:100, Abcam, UK) and goat anti‐rabbit IgG H&L (Alexa Fluor® 647) (ab150079, 1:100, Abcam, UK). Finally, the cytoskeleton and angiogenesis were visualized using a confocal microscope (Leica TCS SP8, Germany). All experiments were performed in triplicate.

### 2.23 Animal experiment grouping and treatment

DFU rats were randomly divided into three groups (*n* = 10 per group): the PBS control group, the NC mimic H‐ADSCs‐Exo group, and the miR‐100‐5p mimic H‐ADSCs‐Exo group. Exo were administered via tail vein injection at a dose of 200 μg per treatment, once per week for 4 consecutive weeks. Foot ulcer healing was monitored weekly, including ulcer size measurements, visual inspection, and photographic documentation.

### 2.24 Detection of miR‐100‐5p expression in rat skin tissue by RT‐qPCR

Total RNA was extracted from rat skin tissue using the TRIzol reagent (15596018, Invitrogen, USA). RT‐qPCR was performed using the TaqMan MicroRNA Assay (4427975, Applied Biosystems, USA), with U6 serving as the internal control. The reaction system and procedures followed the manufacturer's instructions, and amplification was carried out on the ABI 7500 Real‐Time PCR System (Applied Biosystems, USA). Relative expression levels were calculated using the 2^−ΔΔCt^ method, and all experiments were performed in triplicate.

### 2.25 Measurement of skin thickness and hematoxylin and eosin (H&E) staining

Skin thickness around the ulcer area was measured weekly using a vernier caliper, and the changes in thickness for each rat were recorded to assess healing progression. At the end of the experiment, skin tissues were excised, fixed in 10% neutral formalin (Sigma‐Aldrich, USA, Cat. No: HT501128), dehydrated, and embedded in paraffin to prepare 5 μm thick sections. The sections were stained with H&E (Beyotime, China, Cat. No: C0105) and examined for morphological changes under a light microscope (Leica Microsystems, Germany). All experiments were repeated three times.

### 2.26 Immunohistochemistry (IHC) staining

After deparaffinization and rehydration of skin tissue sections, endogenous peroxidase activity was blocked by treatment with 3% hydrogen peroxide (Beyotime, China) for 10 min. Antigen retrieval was performed using citrate buffer (pH 6.0, Beyotime, China) at high temperatures. Primary antibodies against angiogenesis‐related factors VEGF (ab1316, 1:200, Abcam, UK) and CD31 (ab28364, 1:200, Abcam, UK) were applied, and the sections were incubated overnight at 4°C. The following day, the sections were washed with PBS and incubated with HRP‐conjugated secondary antibodies (1:500, Cell Signaling Technology, USA, catalog number: 7076S) at room temperature for 1 h. Color development was performed using DAB substrate (Beyotime, China, catalog number: P0203), followed by hematoxylin counterstaining. The sections were then observed and photographed under a light microscope. All experiments were repeated three times.

### 2.27 Inflammatory cytokine and metabolic marker assays

Serum levels of tumor necrosis factor alpha (TNF‐α) (RTA00, R&D Systems, USA) and interleukin‐6 (IL‐6) (R6000B, R&D Systems, USA) in rats were measured using ELISA kits, following the manufacturer's instructions. Absorbance values were obtained, and cytokine concentrations were calculated accordingly. Blood glucose levels were determined using the glucose oxidase method, with fasting blood samples collected and measured using a glucose meter (Accu‐Chek, Roche, USA). Serum insulin levels were also assessed using an ELISA kit (EZRMI‐13K, Merck Millipore, USA), with absorbance readings used to calculate insulin concentrations. All experiments were performed in triplicate.

### 2.28 Ethical statement and data analysis

The animal experiments in this study were conducted in strict accordance with the Guide for the Care and Use of Laboratory Animals. All experimental procedures were approved by the Institutional Animal Care and Use Committee (IACUC). The animals were handled in an ethically compliant environment, with measures such as proper anesthesia, postoperative care, and pain relief implemented to ensure their health and well‐being. Throughout this study, all procedures involving animals adhered to ethical standards aimed at minimizing pain and discomfort, with humane euthanasia performed when necessary.

For statistical analysis, all data were expressed as mean ± standard deviation (mean ± SD). Differences between groups were assessed using one‐way analysis of variance (ANOVA), with a significance level set at *p* < 0.05. Post hoc analysis for multiple comparisons was performed using Tukey's HSD test. For nonnormally distributed data, nonparametric tests, such as the Mann–Whitney *U* test or the Kruskal–Wallis test, were applied. Time‐dependent data from in vivo experiments were analyzed using repeated‐measures ANOVA to evaluate the impact of time on the results. All statistical analyses were performed using GraphPad Prism 8 (GraphPad software, USA), with a significance threshold of *p* < 0.05, indicating statistically significant differences.

## 3 RESULTS

### 3.1 Isolation and characterization of ADSC‐Exo

ADSCs were successfully isolated from iWAT of adult SD rats. Flow cytometry and differentiation assays confirmed the identity of the cells (Figure S1A), showing high expression of surface markers CD29, CD44, CD90, and CD73, while lacking expression of CD34, CD19, and CD45 (Figure S1B). Additionally, these ADSCs demonstrated the potential for osteogenic and adipogenic differentiation (Figure S1C), consistent with the phenotypic characteristics of mesenchymal stem cells.

Exo were isolated from ADSC culture supernatants using differential centrifugation and UC, followed by characterization. TEM revealed that the isolated Exo displayed a typical spherical vesicle structure with diameters ranging from 30 to 150 nm (Figure S1D). NTA indicated that the majority of Exo had a particle size of approximately 100 nm (Figure S1E). WB analysis confirmed the presence of Exo‐specific markers CD63, CD81, and TSG101, whereas the negative marker calnexin was absent (Figure S1F). These findings confirm the successful isolation and purification of Exo from ADSCs.

### 3.2 Transcriptome sequencing reveals the crucial role of miR‐100‐5p in DFU healing

DFUs are among the most severe complications of diabetes, and conventional treatment methods have shown limited efficacy, underscoring the urgent need for new therapeutic strategies.^1^ In recent years, ADSCs‐Exo have demonstrated significant potential in promoting wound healing. Notably, the miRNA components within these nanoscale vesicles can effectively deliver proteins, RNA, and miRNAs, facilitating intercellular communication and regulating the function of target cells.^23^ Therefore, further exploration of the regulatory mechanisms of ADSCs‐Exo is essential for elucidating their molecular role in wound healing and providing a scientific basis for developing innovative therapeutic approaches.

**FIGURE 1:**
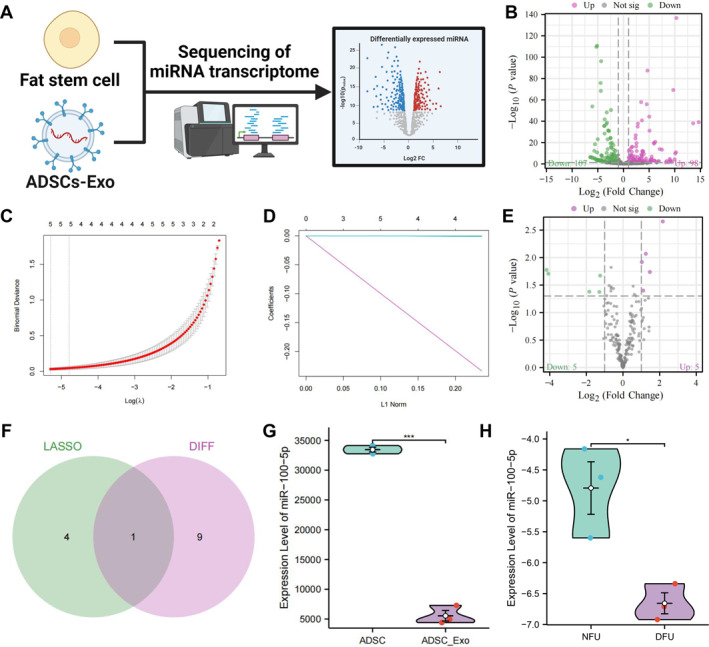
Transcriptome sequencing reveals key miRNAs in Exo that regulate DFU progression. (A) Schematic diagram showing differentially expressed miRNAs between ADSCs and ADSCs‐Exo identified by transcriptome sequencing; (B) volcano plot showing differential miRNA expression between ADSCs and ADSCs‐Exo; (C) LASSO coefficient distribution of differentially expressed genes; (D) selection of optimal parameters (lambda) for the LASSO model; (E) volcano plot showing differential miRNA expression between DFU and NFU (healthy) patient skin samples, *n* = 3; (F) intersection of differentially expressed miRNAs in NFU and DFU samples with features identified by LASSO analysis; (G) transcriptomic expression levels of miR‐100‐5p in ADSCs and ADSCs‐Exo; (H) transcriptomic expression levels of miR‐100‐5p in DFU and NFU samples, *n* = 3. ADSCs‐Exo, adipose‐derived stem cell exosomes; DFU, diabetic foot ulcer; LASSO, least absolute shrinkage and selection operator; NFU, nondiabetic foot ulcers.

To investigate the specific biological functions mediated by ADSCs‐Exo, we conducted miRNA transcriptome sequencing on both ADSCs and ADSCs‐Exo (Figure 1A), using ADSCs as the control group. Differential expression analysis identified 205 differentially expressed miRNAs, with 98 upregulated and 107 downregulated in the Exo (Figure 1B). To further elucidate the functional implications of these differentially expressed miRNAs, we predicted their potential target genes using RNAhybrid and miRanda software (Table S2). We then performed GO and KEGG pathway enrichment analyses using clusterProfiler. GO analysis revealed that the target genes of the differentially expressed miRNAs were primarily enriched in critical biological processes and molecular functions, such as “phosphorylation,” “phosphate metabolic processes,” and “regulation of signal transduction,” all of which play essential roles in cellular signaling and regulation under both physiological and pathological conditions. These processes significantly impact cell growth, differentiation, and metabolism. Additionally, the genes were enriched in molecular functions such as “protein binding,” “ion binding,” and “ATP binding,” suggesting that the differentially expressed miRNAs are closely related to enzymatic activity, signal transduction, and energy metabolism^24^ (Figure S2A). KEGG pathway analysis showed that the target genes of these miRNAs were highly associated with the Wnt and MAPK signaling pathways (Figure S2B). These pathways have been shown to play key roles in the healing process of DFU,^25^ indicating that ADSCs‐Exo may have a regulatory effect on DFU healing.

To further identify key miRNAs, we applied LASSO regression analysis to fit the differentially expressed miRNAs and eliminate redundant features, ultimately identifying five feature factors (Figure 1C,D). In parallel, we analyzed DFU‐related miRNA transcriptome data from the GEO database, identifying 10 differentially expressed miRNAs between DFU and non‐DFU samples (Figure 1E). By intersecting these with the factors identified through LASSO analysis, miR‐100‐5p emerged as the sole overlapping factor (Figure 1F). Previous studies have shown that miR‐100‐5p plays a multifaceted role in regulating key cellular functions such as proliferation, migration, and angiogenesis, processes closely linked to DFU healing.^26^, ^27^ Transcriptome data revealed that miR‐100‐5p levels were significantly lower in ADSCs‐Exo compared to ADSCs (Figure 1G), and similarly, miR‐100‐5p expression was significantly reduced in DFU samples compared to NFU samples (Figure 1H). These findings suggest that the downregulation of miR‐100‐5p may contribute to the development of DFU, with its low expression potentially serving as a barrier to wound healing.

In conclusion, miR‐100‐5p is a key miRNA in Exo that may regulate DFU progression, and its reduced expression could play a critical role in promoting DFU advancement.

### 3.3 H‐ADSCs‐Exo promote DFU healing and reduce IR

In vitro, a hypoxic environment was created by culturing ADSCs under 1% O_2_ to induce hypoxia. H‐ADSCs‐Exo were isolated and purified using differential centrifugation and UC (Figure S3A). TEM analysis revealed that H‐ADSCs‐Exo displayed a typical round vesicle structure with diameters ranging from 30 to 150 nm, similar to N‐ADSCs‐Exo (Figure S3B). NTA showed no significant differences in particle size distribution between H‐ADSCs‐Exo and N‐ADSCs‐Exo, with both having a mean size of approximately 100 nm (Figures S3C and S1E). WB analysis confirmed that both H‐ADSCs‐Exo and N‐ADSCs‐Exo expressed CD63, CD81, and TSG101, while lacking calnexin expression (Figure S3D). These findings confirm the successful isolation and purification of H‐ADSCs‐Exo.

**FIGURE 2:**
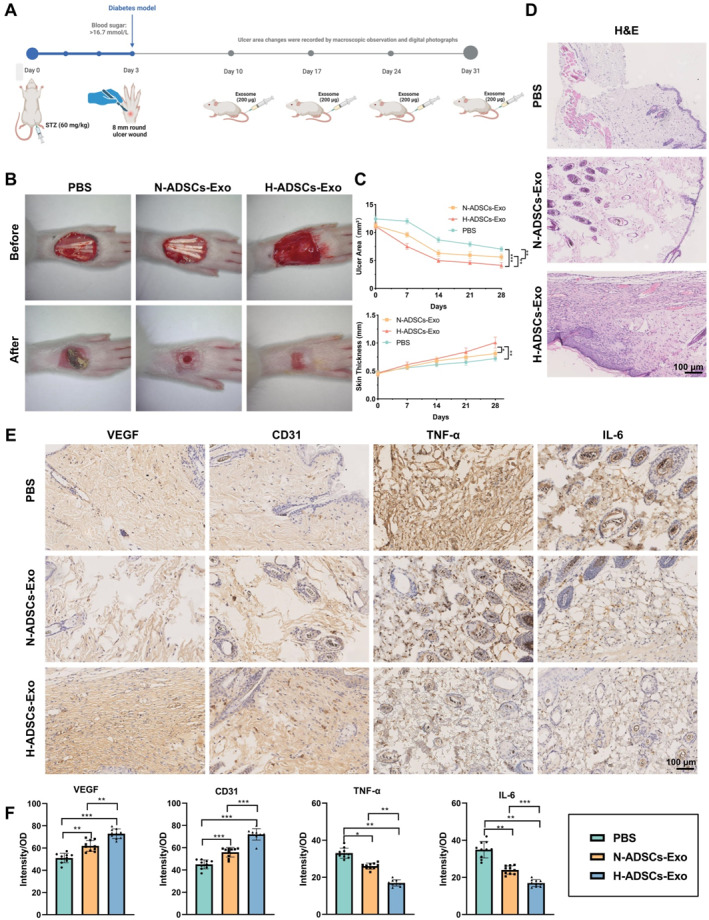
Therapeutic effects of ADSCs‐Exo in a DFU rat model. (A) Schematic diagram illustrating the treatment process of the DFU rat model with ADSCs‐Exo; (B) visual observation and digital image recording of ulcer area changes in the DFU rat model; (C) the wound area and skin thickness were measured using a vernier caliper; (D) hematoxylin and eosin staining of skin tissue morphology in the DFU rat model; (E) IHC analysis of angiogenesis‐related factors VEGF and CD31, and inflammatory factors TNF‐α and IL‐6; (F) quantification of VEGF, CD31, TNF‐α, and IL‐6 expression through IHC. All data are presented as mean ± standard error, 10 rats per group (*n* = 10). Statistical analysis was performed using ANOVA followed by Tukey's post hoc test. ADSCs‐Exo, adipose‐derived stem cell exosomes; DFU, diabetic foot ulcer; IHC, immunohistochemistry; IL‐6, interleukin‐6; TNF‐α, tumor necrosis factor alpha; VEGF, vascular endothelial growth factor. *p < 0.05, **p < 0.01, ***p < 0.001.

The DFU rat model was further established by intraperitoneal injection of STZ at a dose of 60 mg/kg body weight to induce diabetes. After 3 days, blood glucose levels stabilized above 16.7 mmol/L, confirming the successful establishment of the diabetic model. A circular ulcer with a diameter of 8 mm was then created on the rats' feet via surgery. The ulcer area was monitored through visual observation and digital imaging to assess the success of the ulcer model (Figure 2A). The rats were randomly divided into three groups: the PBS control group, the N‐ADSCs‐Exo‐treated group, and the H‐ADSCs‐Exo‐treated group. Exo (200 μg) was administered via tail vein injection once a week for four consecutive weeks. The results showed that the H‐ADSCs‐Exo‐treated group exhibited a significant reduction in ulcer size and an increase in skin thickness (Figure 2B,C). H&E staining and IHC analysis revealed that angiogenesis‐related factors, VEGF and CD31, were significantly upregulated, whereas inflammatory markers, TNF‐α and IL‐6, were notably downregulated in the H‐ADSCs‐Exo‐treated group (Figure 2D–F). These findings suggest that H‐ADSCs‐Exo significantly promote DFU healing.

### 3.4 H‐ADSCs‐Exo deliver miR‐100‐5p to promote cell proliferation and angiogenesis

**FIGURE 3:**
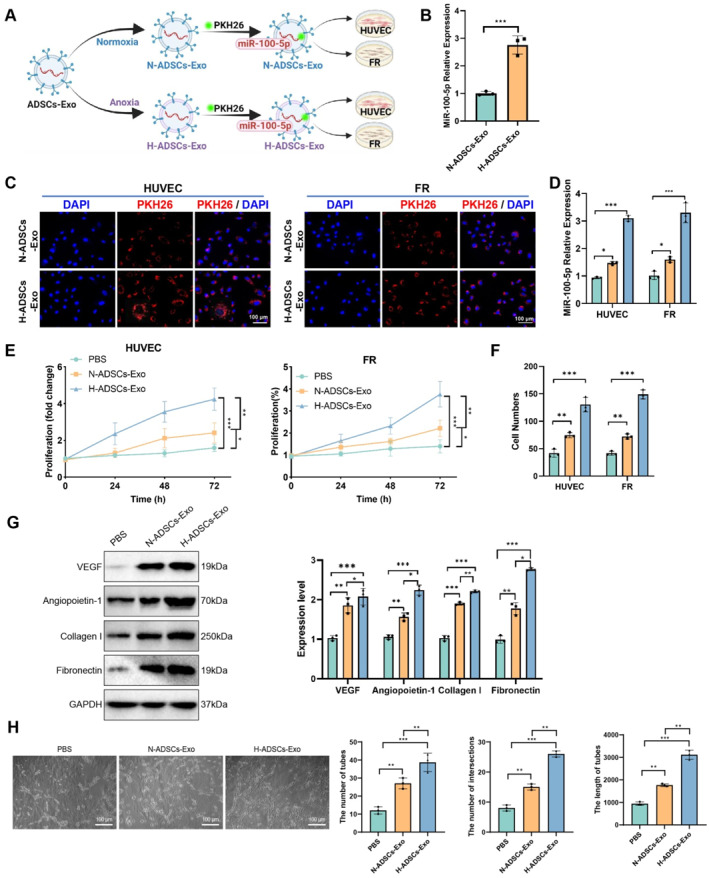
The role of H‐ADSCs‐Exo‐mediated miR‐100‐5p delivery in cell proliferation and angiogenesis. (A) Schematic diagram of H‐ADSCs‐Exo delivering miR‐100‐5p to treat HUVEC and FR cells; (B) RT‐qPCR analysis of miR‐100‐5p expression levels in H‐ADSCs‐Exo and N‐ADSCs‐Exo; (C) confocal microscopy observation of fluorescently labeled H‐ADSCs‐Exo and N‐ADSCs‐Exo uptake in HUVEC and FR cells; (D) RT‐qPCR analysis of miR‐100‐5p expression in HUVEC and FR cells after treatment with H‐ADSCs‐Exo and N‐ADSCs‐Exo; (E) CCK‐8 assay to determine the proliferation rates of HUVEC and FR cells treated with PBS, N‐ADSCs‐Exo, and H‐ADSCs‐Exo; (F) Transwell assay to assess the migration abilities of HUVEC and FR cells treated with PBS, N‐ADSCs‐Exo, and H‐ADSCs‐Exo; (G) Western blot analysis of the expression levels of vascular endothelial growth factor, angiopoietin 1, collagen I, and fibronectin proteins in HUVEC treated with PBS, N‐ADSCs‐Exo, and H‐ADSCs‐Exo; (H) tube formation assay to evaluate the number, length, and branching points of tubular structures formed by HUVEC treated with PBS, N‐ADSCs‐Exo, and H‐ADSCs‐Exo. All data are presented as mean ± standard error, with experiments repeated in triplicate (*n* = 3 biological replicates). Statistical analysis was performed using ANOVA followed by Tukey's post hoc test. FR, fibroblasts; H‐ADSCs‐Exo, hypoxic adipose‐derived stem cell exosomes; HUVEC, human umbilical vein endothelial cells; N‐ADSCs‐Exo, normoxic adipose‐derived stem cell exosomes; RT‐qPCR, quantitative real‐time polymerase chain reaction. *p < 0.05, **p < 0.01, ***p < 0.001.

In this study, to investigate the mechanism by which H‐ADSCs‐Exo deliver miR‐100‐5p to promote cell proliferation and angiogenesis, we designed a multistep validation process (Figure 3A). RT‐qPCR analysis revealed that the expression level of miR‐100‐5p in H‐ADSCs‐Exo was significantly higher than in N‐ADSCs‐Exo (Figure 3B). To further confirm the uptake of ADSCs‐Exo by cells, PKH26‐labeled N‐ADSCs‐Exo and H‐ADSCs‐Exo were incubated with HUVEC and FR. Confocal microscopy showed that the uptake efficiency of H‐ADSCs‐Exo in both cell types was markedly higher than that of N‐ADSCs‐Exo (Figure 3C). RT‐qPCR analysis of miR‐100‐5p expression levels in cells indicated that miR‐100‐5p was significantly more abundant in HUVEC and FR treated with H‐ADSCs‐Exo compared to those treated with N‐ADSCs‐Exo (Figure 3D). These results demonstrate that H‐ADSCs‐Exo has a significant advantage in enhancing the uptake and expression of miR‐100‐5p.

HUVEC and FR cells treated with high glucose were subjected to treatment with PBS, N‐ADSCs‐Exo, and H‐ADSCs‐Exo. Cell proliferation was assessed using the CCK‐8 assay, which revealed that the H‐ADSCs‐Exo treatment group had a significantly higher proliferation rate compared to the other two groups (Figure 3E). Transwell assay results showed that the H‐ADSCs‐Exo group exhibited a marked increase in cell migration capacity (Figure 3F). WB analysis further indicated that protein expression levels of VEGF, angiopoietin 1, collagen I, and fibronectin were significantly higher in the H‐ADSCs‐Exo group than in the N‐ADSCs‐Exo group (Figure 3G). The results showed that Exo treatment from hypoxic conditions significantly enhanced tube formation (length, number, and junctions) compared to Exo treatment from normoxic conditions in HUVEC (Figure 3H). These findings suggest that H‐ADSCs‐Exo, by promoting miR‐100‐5p expression, significantly enhances angiogenesis and cell migration capacity.

### 3.5 Hypoxic Exo promote angiogenesis and cell proliferation via miR‐100‐5p

**FIGURE 4:**
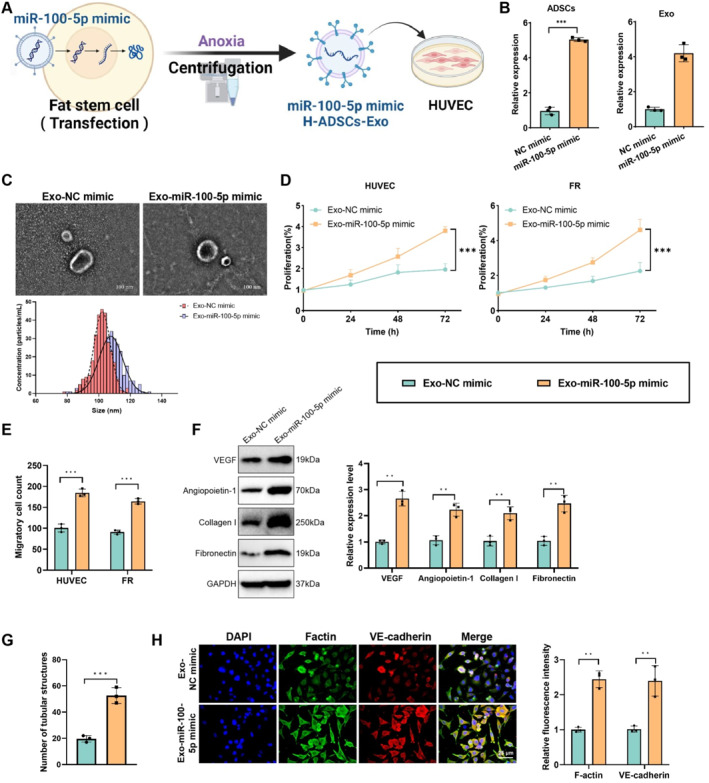
The role of H‐ADSCs‐Exo overexpressing miR‐100‐5p in promoting angiogenesis and cell proliferation. (A) Schematic representation of the procedure for treating HUVEC with H‐ADSCs‐Exo overexpressing miR‐100‐5p; (B) quantitative real‐time polymerase chain reaction analysis of miR‐100‐5p expression levels in H‐ADSCs‐Exo overexpressing miR‐100‐5p mimic and NC mimic; (C) transmission electron microscopy and nanoparticle tracking analysis to characterize the morphology and size distribution of H‐ADSCs‐Exo overexpressing miR‐100‐5p mimic and NC mimic; (D) cell counting kit‐8 assay to measure the proliferation rates of HUVEC and fibroblasts cells treated with Exo overexpressing miR‐100‐5p mimic and NC mimic; (E) Transwell assay to evaluate the migration ability of cells treated with Exo overexpressing miR‐100‐5p mimic and NC mimic; (F) Western blot analysis of the expression levels of vascular endothelial growth factor, angiopoietin 1, collagen I, and fibronectin proteins in HUVEC treated with miR‐100‐5p mimic and NC mimic Exo; (G) tube formation assay to assess the number of tubular structures formed by HUVEC treated with Exo overexpressing miR‐100‐5p mimic and NC mimic; (H) immunofluorescence staining to detect F‐actin and VE‐cadherin expression in HUVEC treated with Exo overexpressing miR‐100‐5p mimic and NC mimic. All data are presented as mean ± standard error, with experiments repeated three times (*n* = 3 biological replicates). Statistical analysis was performed using ANOVA followed by Tukey's post hoc test. H‐ADSCs‐Exo, hypoxic adipose‐derived stem cell exosomes; HUVEC, human umbilical vein endothelial cells. *p < 0.05, **p < 0.01, ***p < 0.001.

The results indicate that, compared to normoxic treatment, Exo from hypoxic conditions showed significantly higher expression of miR‐100‐5p, along with enhanced angiogenesis and cell proliferation. Further investigation explored the effects of overexpressing or knocking down miR‐100‐5p in hypoxic Exo on angiogenesis and cell proliferation (Figure 4A, Figure S4A). Lentiviral vectors were used to overexpress miR‐100‐5p and miR‐NC (control) in H‐ADSCs, and RT‐qPCR was used to verify the transfection efficiency, showing successful overexpression of miR‐100‐5p in ADSCs and their secreted Exo (Figure 4B). The RT‐qPCR results also confirmed that miR‐100‐5p was successfully knocked down in ADSCs and their secreted Exo (Figure S4B). H‐ADSCs‐Exo with overexpression or knockdown of miR‐100‐5p or control Exo were then isolated and purified. TEM and NTA showed no significant differences in the morphology and particle size distribution of Exo between the overexpression, knockdown, and control groups (Figure 4C, Figure S4C).

These Exo were used to treat high‐glucose‐induced HUVEC and FR cells, and cell proliferation was detected using the CCK‐8 assay. The results showed that Exo overexpressing miR‐100‐5p significantly increased cell proliferation in both HUVEC and FR cells compared to miR‐NC Exo‐treated groups (Figure 4D). Knockdown of miR‐100‐5p in Exo resulted in a significant reduction in cell proliferation in both HUVEC and FR cells compared to miR‐NC Exo‐treated groups (Figure S4D). Transwell assays demonstrated that miR‐100‐5p mimic Exo treatment significantly enhanced cell migration (Figure 4E), whereas knockdown of miR‐100‐5p Exo reduced migration ability (Figure S4E). Western blot analysis revealed that Exo overexpressing miR‐100‐5p significantly increased the expression of VEGF, angiopoietin 1, collagen I, and fibronectin proteins compared to miR‐NC Exo‐treated groups (Figure 4F), whereas knockdown of miR‐100‐5p Exo significantly reduced these protein levels compared to the control Exo‐treated group (Figure S4F). Tube formation assays showed that miR‐100‐5p mimic Exo‐treated HUVEC formed significantly more tubular structures than the control group (Figure 4G), whereas knockdown of miR‐100‐5p Exo‐treated cells formed significantly fewer tubular structures than the control group (Figure S4G). Immunofluorescence staining results indicated enhanced expression of F‐actin and VE‐cadherin in miR‐100‐5p mimic Exo‐treated cells, with significant cytoskeletal remodeling and increased angiogenesis (Figure 4H), whereas knockdown of miR‐100‐5p in Exo‐treated cells showed decreased expression of F‐actin and VE‐cadherin, with reduced cytoskeletal remodeling and angiogenesis (Figure S4H). These findings suggest that hypoxic Exo promote angiogenesis and cell proliferation via miR‐100‐5p regulation, playing a crucial role in the healing process of DFU.

### 3.6 miR‐100‐5p Exo promote DFU healing and reduce IR

**FIGURE 5:**
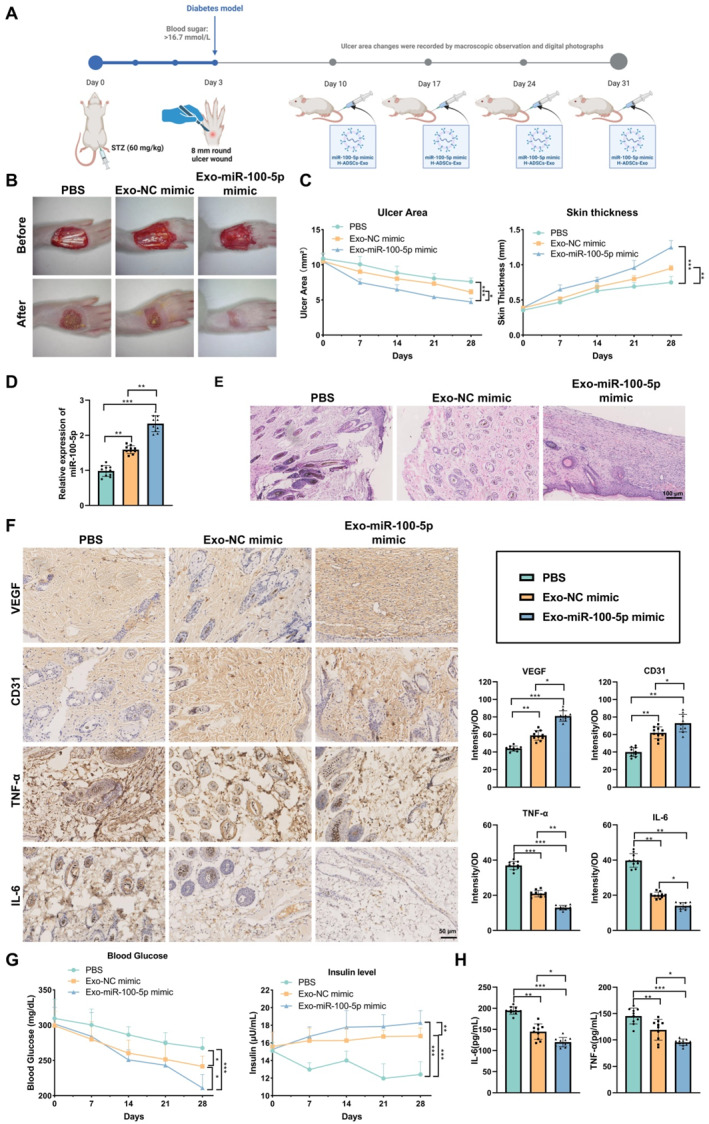
miR‐100‐5p Exo promote healing and reduce inflammatory response in DFU rat models. (A) Schematic diagram illustrating the treatment process of DFU rat models using hypoxic adipose‐derived stem cell exosomes with miR‐100‐5p overexpression; (B) weekly observation and recording of ulcer size changes in DFU rat models; (C) analysis of ulcer area and skin thickness changes; (D) quantitative real‐time polymerase chain reaction detection of miR‐100‐5p expression levels in rat skin tissue; (E) hematoxylin and eosin staining to assess skin tissue morphology; (F) immunohistochemistry detection of angiogenesis‐related factors vascular endothelial growth factor and CD31, and inflammatory factors TNF‐α and IL‐6; (G) measurement of blood glucose and insulin levels; (H) ELISA detection of inflammatory factors IL‐6 and TNF‐α. All data are presented as mean ± standard error, 10 rats per group (*n* = 10). Statistical analysis was performed using ANOVA and Tukey's post hoc test. DFU, diabetic foot ulcer; IL‐6, interleukin‐6; TNF‐α, tumor necrosis factor alpha. *p < 0.05, **p < 0.01, ***p < 0.001.

Rats with DFUs were randomly divided into three groups: the PBS control group, the NC mimic H‐ADSCs‐Exo group, and the miR‐100‐5p mimic H‐ADSCs‐Exo group. Each group received weekly intravenous injections of 200 μg Exo for four consecutive weeks. The healing of the foot ulcers was observed and recorded weekly (Figure 5A). The results showed that, compared to the NC mimic H‐ADSCs‐Exo group, the miR‐100‐5p mimic H‐ADSCs‐Exo treatment group had a significantly smaller ulcer area, whereas the PBS control group had a smaller healing area (Figure 5B‐,C). RT‐qPCR analysis of skin tissue revealed that the miR‐100‐5p expression level in the miR‐100‐5p mimic H‐ADSCs‐Exo group was significantly higher than in the other two groups (Figure 5D).

Through H&E staining and IHC analysis, it was found that the expression of angiogenesis‐related factors VEGF and CD31 was significantly increased in the skin tissues of rats treated with miR‐100‐5p mimic H‐ADSCs‐Exo, whereas the expression of inflammatory factors TNF‐α and IL‐6 was markedly reduced (Figure 5E,F). These results suggest that treatment with miR‐100‐5p mimic Exo effectively promotes angiogenesis and reduces IR. Additionally, blood glucose and insulin assays indicated that rats in the miR‐100‐5p mimic H‐ADSCs‐Exo group had lower blood glucose levels and higher insulin levels compared to the PBS control group (Figure 5G). The ELISA results for inflammatory factors further supported these findings, with significantly lower levels of IL‐6 and TNF‐α in the miR‐100‐5p mimic H‐ADSCs‐Exo group compared to the control group (Figure 5H). Collectively, these data demonstrate that hypoxic miR‐100‐5p mimic Exo significantly enhance DFU healing and improve diabetes‐related parameters.

## 4 DISCUSSION

This study aims to elucidate the mechanism by which H‐ADSCs‐Exo carrying miR‐100‐5p promotes the healing of DFU, providing new strategies and potential targets for DFU treatment. DFU is notoriously difficult to heal due to impaired angiogenesis and chronic inflammation. In recent years, Exo have gained increasing attention for their potential in tissue repair. Previous studies have demonstrated that Exo play a key role in wound healing by mediating intercellular communication and regulating cell proliferation, migration, and angiogenesis.^28^ However, unlike prior research, this study focuses on the role of H‐ADSCs‐Exo and the specific function of miR‐100‐5p. This innovative approach highlights the unique potential of hypoxic Exo in DFU treatment. Compared to N‐ADSCs‐Exo, H‐ADSCs‐Exo exhibit enhanced angiogenic and anti‐inflammatory effects, further validating the significant influence of the hypoxic microenvironment on Exo function.

The application of Exo in tissue repair has been extensively studied, particularly in cardiovascular disease, neural injury, and wound healing.^29^ Previous research has shown that Exo can carry a variety of bioactive molecules, including miRNAs, proteins, and lipids, to regulate the repair processes of damaged tissues.^30^ For instance, studies have demonstrated that bone marrow‐derived mesenchymal stem cell Exo can promote angiogenesis and accelerate cardiac function recovery after myocardial injury.^31^ This study further investigates ADSCs‐Exo, specifically under hypoxic conditions, and how they mediate the healing process of DFU by carrying miR‐100‐5p. Unlike traditional sources and handling of Exo, this research shows that H‐ADSCs‐Exo exhibit greater potential in promoting angiogenesis and inhibiting IRs.

miR‐100‐5p, a critical regulator of gene expression, plays a significant role in various biological processes,^32^ including tumor growth, angiogenesis, and cell proliferation.^33^ However, its role in Exo‐mediated tissue repair remains largely unexplored.^34^ In this study, bioinformatic analysis and LASSO regression identified miR‐100‐5p as a key molecule in the treatment of DFU, further validating its significance in H‐ADSCs‐Exo. Unlike other miRNAs studied, miR‐100‐5p not only promotes angiogenesis but also significantly reduces IRs by regulating several inflammatory factors, such as TNF‐α and IL‐6. These findings provide theoretical support for future miRNA‐targeted therapies for DFU and other chronic wounds.

In this study, angiogenesis and IR regulation were identified as key factors in the healing of DFU. Analysis of the H‐ADSCs‐Exo‐treated DFU rat model revealed a significant upregulation of angiogenesis markers, such as VEGF and CD31, and a marked reduction in inflammatory factors, including TNF‐α and IL‐6. Compared to previous research, this study not only confirms the role of Exo in angiogenesis but also highlights the potential of H‐ADSCs‐Exo to accelerate wound healing through dual mechanisms. In contrast, prior literature has paid limited attention to the impact of the hypoxic microenvironment on Exo function. Our findings demonstrate the importance of this factor in Exo‐mediated regulation of both angiogenesis and IR, addressing a critical gap in the field.

In vitro experiments showed that H‐ADSCs‐Exo‐treated endothelial cells and FRs exhibited significantly enhanced proliferation, migration, and angiogenesis, further confirming the positive role of H‐ADSCs‐Exo in tissue repair. In contrast, the N‐ADSCs‐Exo and PBS control groups demonstrated limited therapeutic effects, particularly in terms of cell proliferation and migration, where notable differences were observed. Additionally, the miR‐100‐5p‐overexpressing H‐ADSCs‐Exo‐treated group showed a stronger upregulation of angiogenesis and fibrosis markers, further validating the central role of miR‐100‐5p in H‐ADSCs‐Exo. Unlike previous studies using N‐ADSCs‐Exo, this study simulates the hypoxic microenvironment of DFU, providing a more realistic experimental basis for exploring the therapeutic potential of H‐ADSCs‐Exo.

Despite the promising findings of this study, there are several limitations. First, although the therapeutic potential of H‐ADSCs‐Exo was validated through both in vivo and in vitro experiments, the precise molecular mechanisms remain to be fully elucidated. Additionally, this study only used a single dose of H‐ADSCs‐Exo, and the effects of different doses of H‐ADSCs‐Exo on DFU healing have not been confirmed. Second, the animal model used in this study may not entirely replicate the complex pathological conditions in human patients. Therefore, future studies should aim to validate these findings in larger‐scale preclinical trials. Additionally, the isolation and purification process for Exo in this study is relatively complex, highlighting the need for more efficient and simplified methods for Exo extraction and application in future research.

Overall, this study is the first to demonstrate the potential of H‐ADSCs‐Exo carrying miR‐100‐5p to accelerate DFU healing by promoting angiogenesis and reducing IR. This research offers novel strategies and potential therapeutic targets for the treatment of DFU, with significant scientific and clinical implications. Future studies should focus on optimizing Exo extraction techniques, exploring their potential in other chronic wounds and tissue injuries, and conducting multicenter clinical trials to advance the clinical translation of this technology.

## 5 CONCLUSION

**FIGURE 6:**
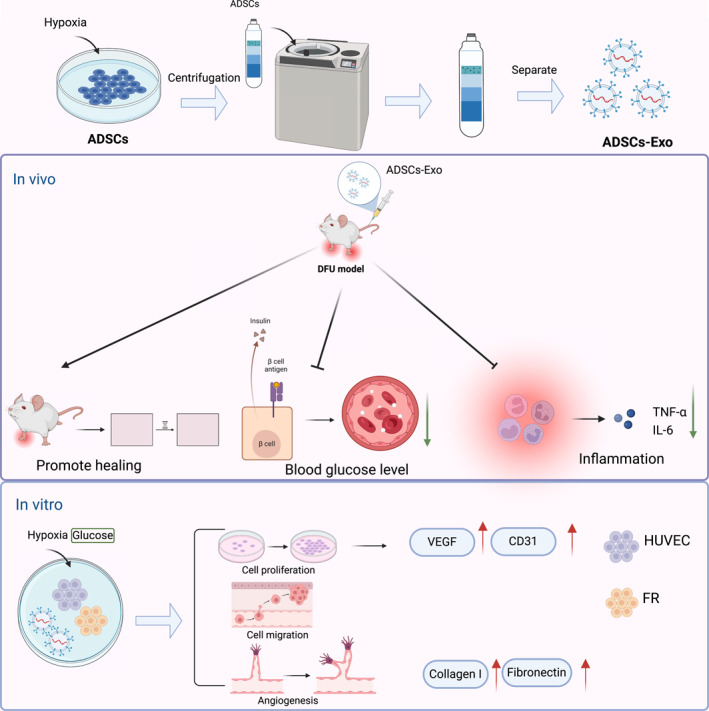
miR‐100‐5p Exo promote diabetic foot ulcer healing by regulating angiogenesis and inflammation.

This study demonstrates that H‐ADSCs‐Exo can deliver miR‐100‐5p, significantly promoting the healing of DFUs. Through in vitro and in vivo experiments, we observed a marked upregulation of miR‐100‐5p expression in H‐ADSCs‐Exo, which exhibited superior cellular uptake and enhanced functionality. The miR‐100‐5p‐loaded Exo significantly promoted angiogenesis and cell proliferation in HUVEC and FR, while reducing IRs, and showed promising therapeutic effects in a DFU rat model (Figure 6).

This study offers new insights and potential therapeutic targets for DFU treatment. Utilizing H‐ADSCs‐Exo to deliver miR‐100‐5p can significantly enhance angiogenesis, cell migration, and proliferation, thereby accelerating the DFU healing process. Additionally, miR‐100‐5p H‐ADSCs‐Exo therapy significantly reduced inflammatory cytokine levels and improved diabetes‐related parameters, highlighting its potential clinical application. Future therapeutic strategies could be developed based on these findings to create more efficient and targeted DFU treatments. Despite the promising results, this study has certain limitations. First, the research primarily focuses on rat models and in vitro cell experiments, lacking clinical validation. Second, the precise molecular mechanisms of Exo‐mediated miR‐100‐5p regulation of angiogenesis and IRs remain to be fully elucidated. Future studies should prioritize clinical trials to confirm the safety and efficacy of miR‐100‐5p Exo in humans. Moreover, optimizing Exo production and enhancing their targeted delivery efficiency is crucial for achieving better therapeutic outcomes.

## AUTHOR CONTRIBUTIONS

Hong Liu contributed to the study conception and design, performed experiments, and analyzed data. Fei Hao supervised the project, provided critical intellectual input, and contributed to manuscript writing and revision. Bangtao Chen was responsible for data interpretation, experimental validation, and manuscript editing. All authors read and approved the final manuscript.

## CONFLICT OF INTEREST STATEMENT

The authors declare no conflicts of interest.

## ETHICS STATEMENT

This study was conducted in strict accordance with ethical guidelines. The animal experiments were approved by the Ethics Committee for Animal Research at the Chongqing University Three Gorges Hospital, with the approval number SXYYDW2024‐004. No human clinical subjects were involved in this study, and therefore, clinical ethical approval was not required.

## CONSENT FOR PUBLICATION

All authors have read and approved the final version of the manuscript and consent to its publication. No individual or identifiable data are included in this study.

## Supporting information

Figures S1–S4

Table S1

Table S2

## Data Availability

All data can be provided as needed.

## References

[1] McDermott, K. , M. Fang , A. J. M. Boulton , E. Selvin , and C. W. Hicks . 2022. “Etiology, Epidemiology, and Disparities in the Burden of Diabetic Foot Ulcers.” Diabetes Care 46(1): 209–221. 10.2337/dci22-0043. PMC9797649 36548709

[2] Okonkwo, U. , and L. DiPietro . 2017. “Diabetes and Wound Angiogenesis.” International Journal of Mathematics and Statistics 18(7): 1419. 10.3390/ijms18071419. PMC5535911 28671607

[3] Del Cuore, A. , R. M. Pipitone , A. Casuccio , M. M. Mazzola , M. G. Puleo , G. Pacinella , R. Riolo , et al. 2023. “Metabolic Memory in Diabetic Foot Syndrome (DFS): MICRO‐RNAS, Single Nucleotide Polymorphisms (SNPs) Frequency and Their Relationship with Indices of Endothelial Function and Adipo‐Inflammatory Dysfunction.” Cardiovascular Diabetology 22(1): 148. 10.1186/s12933-023-01880-x. 37365645 PMC10294440

[4] Armstrong, D. G. , M. A. Swerdlow , A. A. Armstrong , M. S. Conte , W. V. Padula , and S. A. Bus . 2020. “Five Year Mortality and Direct Costs of Care for People with Diabetic Foot Complications Are Comparable to Cancer.” Journal of Foot and Ankle Research 13(1). 10.1186/s13047-020-00383-2. PMC7092527 32209136

[5] Dardari, D. , S. Franc , G. Charpentier , L. Orlando , E. Bobony , M. Bouly , I. Xhaard , et al. 2023. “Hospital Stays and Costs of Telemedical Monitoring versus Standard Follow‐Up for Diabetic Foot Ulcer: an Open‐Label Randomised Controlled Study.” The Lancet Regional Health ‐ Europe 32: 100686. 10.1016/j.lanepe.2023.100686. 37520145 PMC10384180

[6] Zhang, Z. , and L. Lv . 2015. “Effect of Local Insulin Injection on Wound Vascularization in Patients with Diabetic Foot Ulcer.” Experimental and Therapeutic Medicine 11(2): 397–402. 10.3892/etm.2015.2917. 26893621 PMC4734220

[7] Yang, L. , G. C. Rong , and Q. N. Wu . 2022. “Diabetic Foot Ulcer: Challenges and Future.” World Journal of Diabetes 13(12): 1014–1034. 10.4239/wjd.v13.i12.1014. 36578870 PMC9791573

[8] Yu, C. O. L. , K. S. Leung , J. L. Jiang , T. B. Y. Wang , S. K. H. Chow , and W. H. Cheung . 2017. “Low‐Magnitude High‐Frequency Vibration Accelerated the Foot Wound Healing of N5‐Streptozotocin‐Induced Diabetic Rats by Enhancing Glucose Transporter 4 and Blood Microcirculation.” Scientific Reports 7(1): 11631. 10.1038/s41598-017-11934-2. 28912573 PMC5599683

[9] Wang, X. , L. Meng , J. Zhang , L. Zou , Z. Jia , X. Han , L. Zhao , et al. 2023. “Identification of Angiogenesis‐Related Genes in Diabetic Foot Ulcer Using Machine Learning Algorithms.” Heliyon 9(12): e23003. 10.1016/j.heliyon.2023.e23003. 38076120 PMC10703730

[10] Li, M. 2021. “Guidelines and Standards for Comprehensive Clinical Diagnosis and Interventional Treatment for Diabetic Foot in China (Issue 7.0).” Journal of Interventional Medicine 4(3): 117–129. 10.1016/j.jimed.2021.07.003. 34805959 PMC8562298

[11] Pal, D. , P. Das , P. Mukherjee , S. Roy , S. Chaudhuri , S. S. Kesh , D. Ghosh , and S. K. Nandi . 2024. “Biomaterials‐Based Strategies to Enhance Angiogenesis in Diabetic Wound Healing.” ACS Biomaterials Science & Engineering 10(5): 2725–2741. 10.1021/acsbiomaterials.4c00216. 38630965

[12] Guo, E. , L. Wang , J. Wu , and Q. Chen . 2025. “Exosomes from MicroRNA‐125b‐Modified Adipose‐Derived Stem Cells Promote Wound Healing of Diabetic Foot Ulcers.” CSCR 20(4): 409–420. 10.2174/011574888x287173240415050555. 38659271

[13] Liang, Z.‐H. , N.‐F. Pan , S.‐S. Lin , Z.‐Y. Qiu , P. Liang , J. Wang , Z. Zhang , and Y.‐C. Pan . 2022. “Exosomes from Mmu_circ_0001052‐Modified Adipose‐Derived Stem Cells Promote Angiogenesis of DFU via miR‐106a‐5p and FGF4/p38MAPK Pathway.” Stem Cell Research & Therapy 13(1): 336. 10.1186/s13287-022-03015-7. 35870977 PMC9308214

[14] Jing, S. , H. Li , and H. Xu . 2023. “Mesenchymal Stem Cell Derived Exosomes Therapy in Diabetic Wound Repair.” IJN 18: 2707–2720. 10.2147/ijn.s411562. 37250470 PMC10216860

[15] Huang, H. , W. Zhu , Z. Huang , D. Zhao , L. Cao , and X. Gao . 2023. “Adipose‐derived Stem Cell Exosome NFIC Improves Diabetic Foot Ulcers by Regulating miR‐204‐3p/HIPK2.” Journal of Orthopaedic Surgery and Research 18(1): 687. 10.1186/s13018-023-04165-x. 37710299 PMC10503042

[16] Che, D. , X. Xiang , J. Xie , Z. Chen , Q. Bao , and D. Cao . 2024. “Exosomes Derived from Adipose Stem Cells Enhance Angiogenesis in Diabetic Wound via miR‐146a‐5p/JAZF1 Axis.” Stem Cell Rev and Rep 20(4): 1026–1039. 10.1007/s12015-024-10685-8. 38393667 PMC11087353

[17] Dong, J. , B. Wu , and W. Tian . 2023. “Exosomes Derived from Hypoxia‐Preconditioned Mesenchymal Stem Cells (hypoMSCs‐Exo): Advantages in Disease Treatment.” Cell and Tissue Research 392(3): 621–629. 10.1007/s00441-023-03758-6. 36781483

[18] Wei, J. T. , T. He , K. Shen , Z. G. Xu , J. T. Han , and X.‐K. Yang . 2024. “Adipose Stem Cell‐Derived Exosomes in the Treatment of Wound Healing in Preclinical Animal Models: a Meta‐Analysis.” Burns & Trauma 12. 10.1093/burnst/tkae025. PMC11298109 39099759

[19] Liu, P. , L. Qin , C. Liu , J. Mi , Q. Zhang , S. Wang , D. Zhuang , et al. 2022. “Exosomes Derived from Hypoxia‐Conditioned Stem Cells of Human Deciduous Exfoliated Teeth Enhance Angiogenesis via the Transfer of Let‐7f‐5p and miR‐210‐3p.” Frontiers in Cell and Developmental Biology 10. 10.3389/fcell.2022.879877. PMC9086315 35557954

[20] Gao, H. , Z. Yu , Y. Li , and X. Wang . 2021. “miR‐100‐5p in Human Umbilical Cord Mesenchymal Stem Cell‐Derived Exosomes Mediates Eosinophilic Inflammation to Alleviate Atherosclerosis via the FZD5/Wnt/& Beta;‐Catenin Pathway.” ABBS 53(9): 1166–1176. 10.1093/abbs/gmab093. 34254638

[21] Emad, N. A. , J. Pandit , A. Ali , A. Rathee , P. Solanki , K. Imtiyaz , M. M. A. Rizvi , M. Aqil , M. A. Khan , and Y. Sultana . 2025. “Beeswax‐based Nanoconstructs Enriched Dual Responsive Hydrogel for Diabetic Foot Ulcers in Streptozotocin‐Induced Diabetic Rats.” International Journal of Biological Macromolecules 288: 138500. 10.1016/j.ijbiomac.2024.138500. 39647739

[22] Kandhare, A. D. , P. Ghosh , and S. L. Bodhankar . 2014. “Naringin, a Flavanone Glycoside, Promotes Angiogenesis and Inhibits Endothelial Apoptosis through Modulation of Inflammatory and Growth Factor Expression in Diabetic Foot Ulcer in Rats.” Chemico‐Biological Interactions 219: 101–112. 10.1016/j.cbi.2014.05.012. 24880026

[23] Li, X. , X. Xie , W. Lian , R. Shi , S. Han , H. Zhang , L. Lu , and M. Li . 2018. “Exosomes from Adipose‐Derived Stem Cells Overexpressing Nrf2 Accelerate Cutaneous Wound Healing by Promoting Vascularization in a Diabetic Foot Ulcer Rat Model.” Experimental & Molecular Medicine 50(4): 1–14. 10.1038/s12276-018-0058-5. PMC5938041 29651102

[24] Theocharidis, G. , D. Baltzis , M. Roustit , A. Tellechea , S. Dangwal , R. S. Khetani , B. Shu , et al. 2020. “Integrated Skin Transcriptomics and Serum Multiplex Assays Reveal Novel Mechanisms of Wound Healing in Diabetic Foot Ulcers.” Diabetes 69(10): 2157–2169. 10.2337/db20-0188. 32763913 PMC7506837

[25] Chen, J. , Y. Liu , J. Zhang , Y. Yang , H. Liang , T. Li , L. Yan , L. Zhou , L. Shan , and H. Wang . 2023. “External Application of Human Umbilical Cord‐Derived Mesenchymal Stem Cells in Hyaluronic Acid Gel Repairs Foot Wounds of Types I and II Diabetic Rats through Paracrine Action Mode.” Stem Cells Translational Medicine 12(10): 689–706. 10.1093/stcltm/szad050. 37639574 PMC10552688

[26] Wu, J. , L. Kuang , C. Chen , J. Yang , W. N. Zeng , T. Li , H. Chen , et al. 2019. “miR‐100‐5p‐abundant Exosomes Derived from Infrapatellar Fat Pad MSCs Protect Articular Cartilage and Ameliorate Gait Abnormalities via Inhibition of mTOR in Osteoarthritis.” Biomaterials 206: 87–100. 10.1016/j.biomaterials.2019.03.022. 30927715

[27] Wang, K. , S. Liufu , Z. Yu , X. Xu , N. Ai , X. Li , X. Liu , et al. 2023. “miR‐100‐5p Regulates Skeletal Muscle Myogenesis through the Trib2/mTOR/S6K Signaling Pathway.” International Journal of Mathematics and Statistics 24(10): 8906. 10.3390/ijms24108906. PMC10218945 37240251

[28] Ren, S. , J. Chen , J. Guo , Y. Liu , H. Xiong , B. Jing , X. Yang , et al. 2022. “Exosomes from Adipose Stem Cells Promote Diabetic Wound Healing through the eHSP90/LRP1/AKT Axis.” Cells 11(20): 3229. 10.3390/cells11203229. 36291096 PMC9600018

[29] Bhaskara, M. , O. Anjorin , and M. Wang . 2023. “Mesenchymal Stem Cell‐Derived Exosomal microRNAs in Cardiac Regeneration.” Cells 12(24): 2815. 10.3390/cells12242815. 38132135 PMC10742005

[30] Qing, L. , H. Chen , J. Tang , and X. Jia . 2018. “Exosomes and Their MicroRNA Cargo: New Players in Peripheral Nerve Regeneration.” Neurorehabilitation and Neural Repair 32(9): 765–776. 10.1177/1545968318798955. 30223738 PMC6146407

[31] Zhu, Z. , P. Zhu , X. Fan , X. Mo , and X. Wu . 2023. “Mesenchymal Stem Cell‐Derived Exosomes: a Possible Therapeutic Strategy for Repairing Heart Injuries.” Frontiers in Cell and Developmental Biology 11. 10.3389/fcell.2023.1093113. PMC10348815 37457298

[32] Chen, C. , C. Yang , X. Tian , Y. Liang , S. Wang , X. Wang , Y. Shou , et al. 2023. “Downregulation of miR‐100‐5p in Cancer‐associated Fibroblast‐derived Exosomes Facilitates Lymphangiogenesis in Esophageal Squamous Cell Carcinoma.” Cancer Medicine 12(13): 14468–14483. 10.1002/cam4.6078. 37184125 PMC10358253

[33] Xing, X. , S. Han , Z. Li , and Z. Li . 2020. “Emerging Role of Exosomes in Craniofacial and Dental Applications.” Theranostics 10(19): 8648–8664. 10.7150/thno.48291. 32754269 PMC7392016

[34] Chen, G. , H. Chen , X. Zeng , and W. Zhu . 2022. “Stem Cell‐Derived Exosomal Transcriptomes for Wound Healing.” Frontiers in Surgery 9. 10.3389/fsurg.2022.933781. PMC9417542 36034367

