# Supplementary material for: Hypoxic adipose‐derived stem cell exosomes as carriers of miR‐100‐5p to enhance angiogenesis and suppress inflammation in diabetic foot ulcers
Source: J Cell Commun Signal. 2025 Jun 27;19(3):e70018. doi: 10.1002/ccs3.70018 (PMC12204848; doi:10.1002/ccs3.70018)
Supplement: Supplementary file 1 — Figures S1–S4 [file CCS3-19-e70018-s001.docx]

**
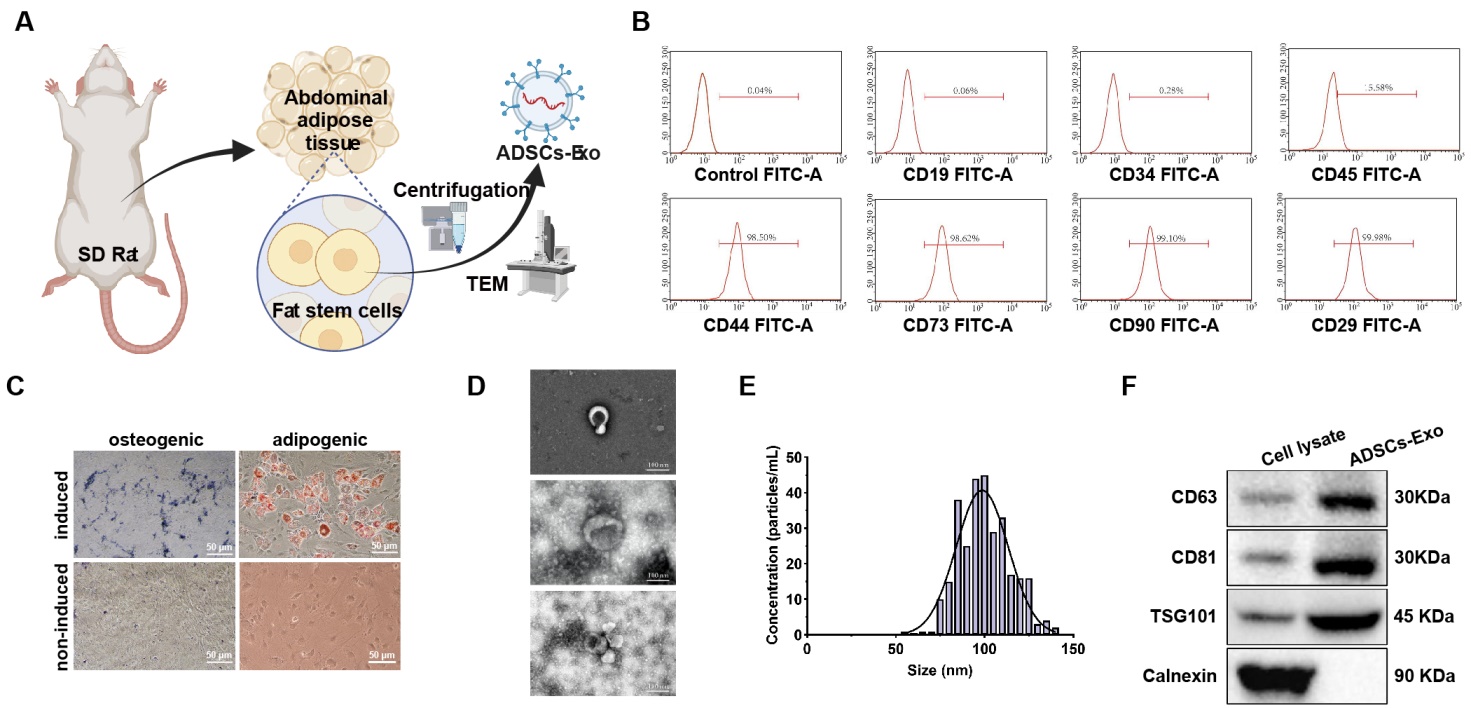
**

**Figure S1. Isolation and Characterization of ADSCs and ADSCs-Exo.**

Note:(A) Schematic diagram illustrating the isolation and characterization process of ADSCs-Exo; (B) Flow cytometry analysis of ADSC surface markers; (C) Oil Red O staining after 14 days of adipogenic induction and alkaline phosphatase staining after 9 days of osteogenic induction to evaluate the differentiation potential of ADSCs (bar = 50 μm); (D) TEM observation of ADSC-Exo morphology and structure (bar = 100 nm); (E) NTA analysis of Exo particle size distribution; (F) WB analysis of specific marker proteins CD63, CD81, TSG101, and Calnexin in ADSCs-Exo. Experiments repeated three times (n=3 biological replicates).

**
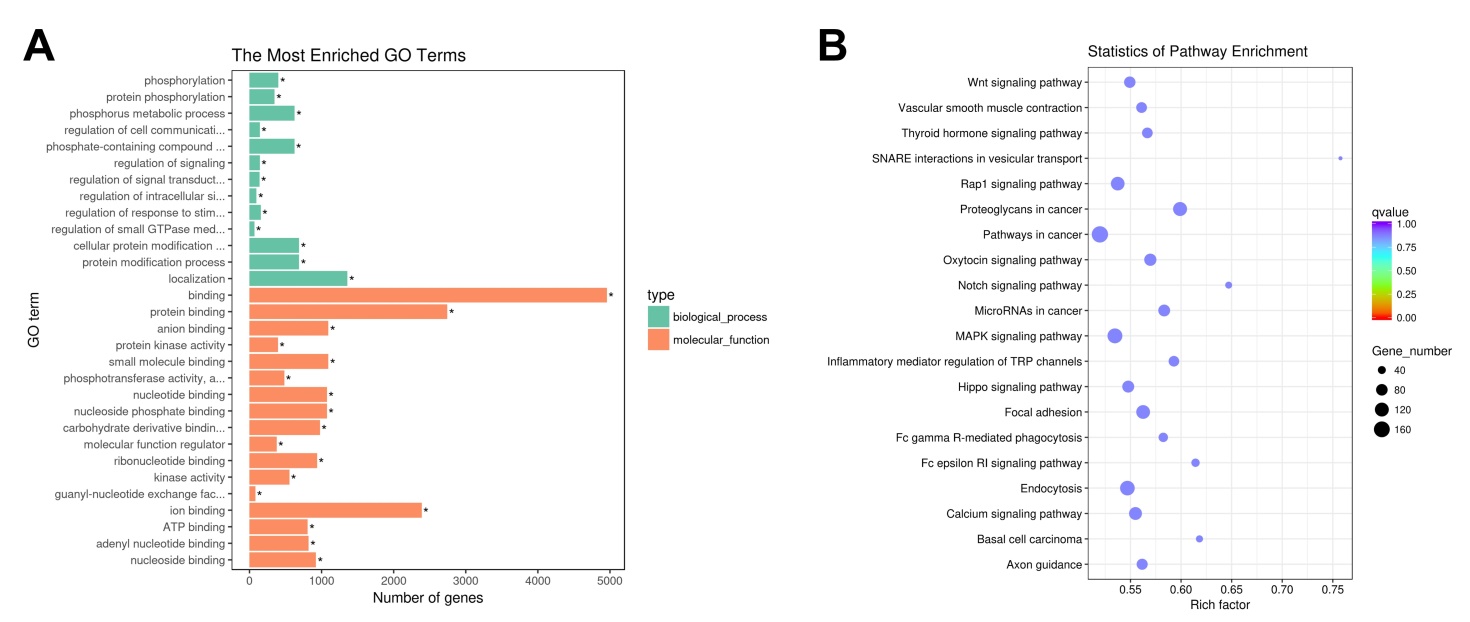
**

**Figure S2. Target Gene Enrichment Analysis of Differentially Expressed miRNAs in ADSCs and ADSCs-Exo.**

Note: (A) GO enrichment analysis of target genes of differentially expressed miRNAs in ADSCs and ADSCs-Exo; (B) KEGG enrichment analysis of target genes of differentially expressed miRNAs in ADSCs and ADSCs-Exo.

**
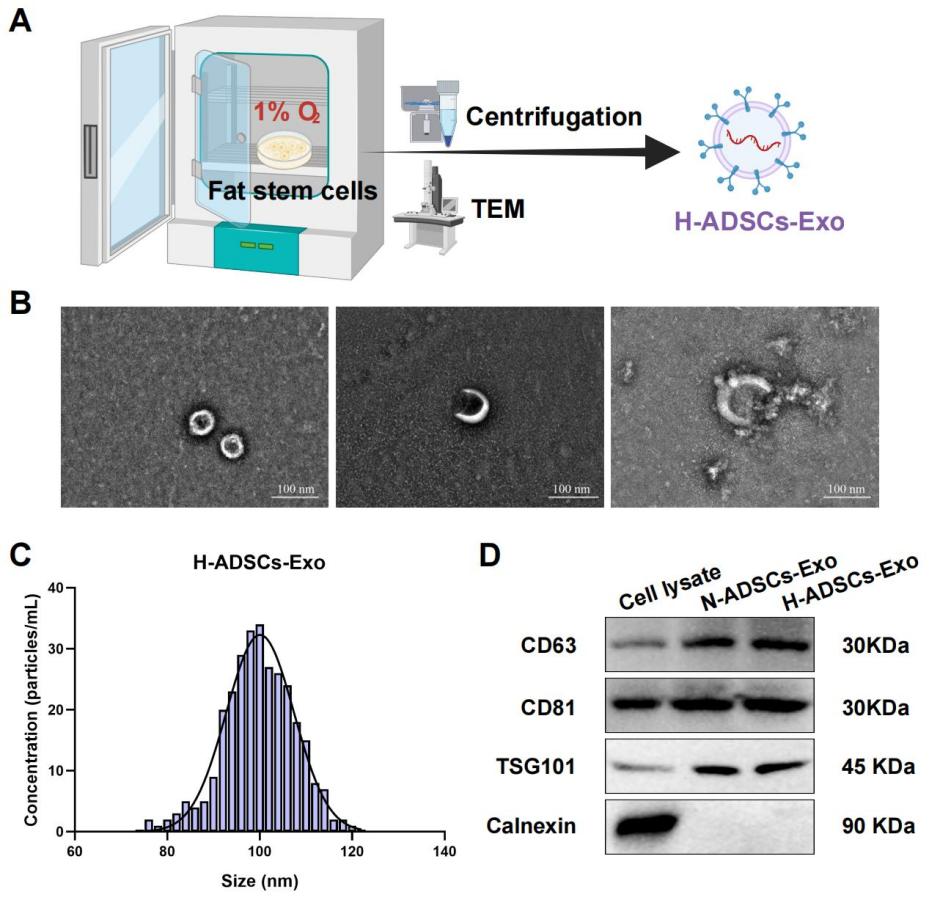
**

**Figure S3. Isolation and Characterization of H-ADSCs-Exo.**

Note: (A) Schematic diagram of the isolation and characterization process of H-ADSCs-Exo; (B) TEM images showing the morphology and structure of H-ADSCs-Exo(bar=100 nm); (C) NTA analysis displaying the size distribution of H-ADSCs-Exo; (D) WB analysis demonstrating the expression of CD63, CD81, TSG101, and Calnexin in ADSCs-Exo. Experiments repeated three times (n=3 biological replicates).

**
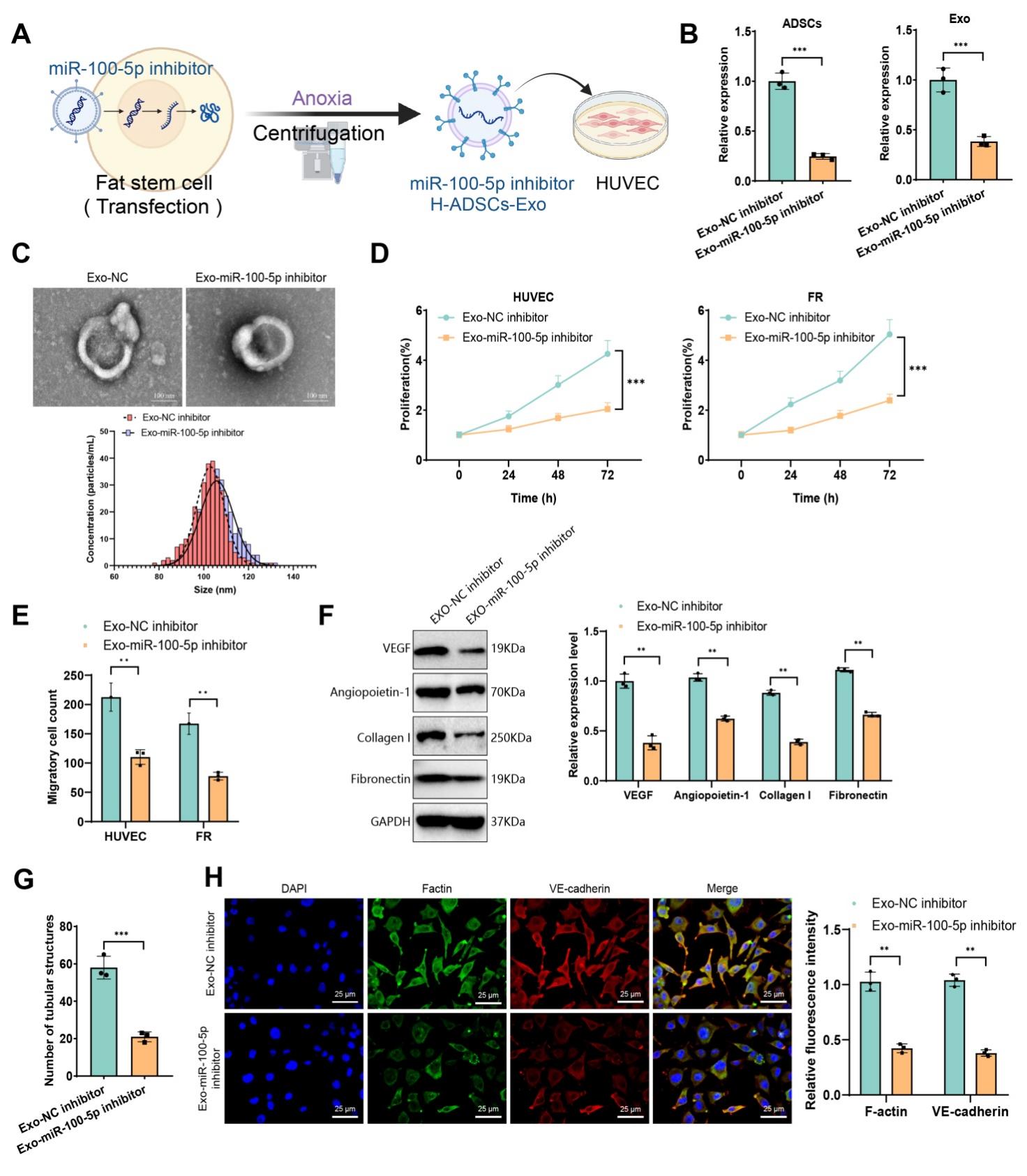
**

**Figure S4. The role of hypoxic Exo with knockdown of miR-100-5p in preventing angiogenesis and cell proliferation.**

Note: (A) Schematic diagram of the process for treating HUVEC cells with H-ADSCs-Exo that have miR-100-5p knockdown; (B)RT-qPCR analysis of miR-100-5p expression in hypoxic ADSCs and their secreted Exo, treated with miR-100-5p inhibitor and NC inhibitor; (C)TEM and NTA characterization of the morphology and particle size distribution of H-ADSCs-Exo treated with miR-100-5p inhibitor and NC inhibitor; (D) CCK-8 assay to detect cell proliferation rate of HUVEC and FR cells treated with miR-100-5p inhibitor and NC inhibitor Exo; (E) Transwell assay to assess cell migration ability in HUVEC and FR cells treated with miR-100-5p inhibitor and NC inhibitor Exo; (F) Western Blot analysis of VEGF, Angiopoietin-1, Collagen I, and Fibronectin protein expression in HUVEC cells treated with miR-100-5p inhibitor and NC inhibitor Exo; (G)Tube formation assay to evaluate the number of tubular structures formed by HUVEC cells treated with miR-100-5p inhibitor and NC inhibitor Exo; (H) Immunofluorescence staining to detect F-actin and VE-cadherin expression in HUVEC cells treated with miR-100-5p inhibitor and NC inhibitor Exo. Data are presented as mean ± SE, with experiments performed in triplicate (n=3 biological replicates). Statistical analysis was conducted using ANOVA followed by Tukey's post-hoc test. * indicates *p* < 0.05, ** indicates *p* < 0.01, and *** indicates *p* < 0.001.
