# Supplementary material for: Hypoxic adipose‐derived stem cell exosomes as carriers of miR‐100‐5p to enhance angiogenesis and suppress inflammation in diabetic foot ulcers
Source: J Cell Commun Signal. 2025 Jun 27;19(3):e70018. doi: 10.1002/ccs3.70018 (PMC12204848; doi:10.1002/ccs3.70018)
Supplement: Supplementary file 2 — Table S1 [file CCS3-19-e70018-s002.docx]

**Table S1. qRT-PCR primer sequences.**

| **Gene Name** | **Sequences (5'-3')** |
| --- | --- |
| **miR-100-5p Forward** | AACCCCGTAGATCCGAACUUGTG |
| **miR-100-5p Reverse** | TaqMan microRNA assay kit Universal reverse primers |
| U6 **Forward** | CTCGCTTCGGCAGCACA |
| U6 **Reverse** | AACGCTTCACGAATTTGCGT |
